# Supplementary material for: Plastid ancestors lacked a complete Entner-Doudoroff pathway, limiting plants to glycolysis and the pentose phosphate pathway
Source: Nat Commun. 2024 Feb 6;15:1102. doi: 10.1038/s41467-024-45384-y (PMC10847513; doi:10.1038/s41467-024-45384-y)
Supplement: Supplementary file 1 — Supplementary Information [file 41467_2024_45384_MOESM1_ESM.pdf]

Supplementary information file

Evans et al (2024) "Plastid ancestors lacked a complete Entner-Doudoroff pathway, limiting plants to glycolysis and the pentose phosphate pathway"

Supplementary table 1. List of organisms used for phylogenetic analysis

Supplementary table 2. Occurrence of EDD homologs in genomes of plastid sister groups

Supplementary table 3. PCR primers used in this study.

Supplementary figure 1. Amino acid alignment of representative DHAD and EDD sequences

Supplementary figure 2. Species phylogeny of the Cyanobacteriota as previously reported by Moore et al.

Supplementary figure 3. SDS-PAGE analysis of purified preparations of dihydroxy acid dehydratase (DHAD), 6-phosphogluconate dehydratase (EDD) and 2-keto-3-deoxy-6-phosphogluconate aldolase (EDA).

Supplementary figure 4. Analytical detection of 2-keto-3-deoxygluconate-6-phosphate (KDPG) by liquid chromatography – tandem mass spectrometry (LC-MS/MS).

Supplementary figure 5. In vitro enzyme assay of purified soybean 2-keto-3-deoxygluconate-6-phosphate aldolase (GmEDA) showing aldolase activity towards substrates of the Calvin-Benson Cycle.

Supplementary figure 6. MS/MS product ion spectra of important metabolites used in this study.

Supplementary references

Supplementary table 1. List of organisms used for phylogenetic analysis

Organism	Phylum	Source	Sequence ID	Gene name
Escherichia coli DH1	Bacteria	[1]	ACX39449.1	EDD
Rhizobium leguminosarum	Bacteria	[1]	WP_116272381.1	EDD
Zymomonas mobilis	Bacteria	[2]	AEH62744.1	EDD
Salmonella enterica	Bacteria	[1]	AGS29956.1	EDD
Acinetobacter baumannii	Bacteria	[1]	WP_031976293.1	EDD
Pseudomonas fluorescens	Bacteria	[1]	SNY07451.1	EDD
Sinorhizobium meliloti	Bacteria	[1]	SDY78282.1	EDD
Agrobacterium tumefaciens	Bacteria	[1]	KJX89456.1	EDD
Xanthomonas campestris	Bacteria	[1]	CEM58200.1	EDD
Cereibacter sphaeroides KD131	Bacteria	[1]	ACM00817.1	EDD
Dinoroseobacter shibae	Bacteria	[1]	ABV93511.1	EDD
Chloroflexota bacterium	Bacteria	[1]	TME26042.1	EDD
Anaerolineae bacterium	Bacteria	[1]	CAG0952899.1	EDD
Phaeobacter gallaeciensis	Bacteria	[1]	AHD08411.1	EDD
Acidiphilium cryptum JF-5	Bacteria	[1]	ABQ29690.1	EDD
Empedobacter haloabium	Bacteria	[1]	TXE25295.1	EDD
Cryomorphaceae bacterium	Bacteria	[1]	MCF8220629.1	EDD
Fluviicola sp.	Bacteria	[1]	PHR35100.1	EDD
Luteimonas aquatica	Bacteria	[1]	WP_242107788.1	EDD
Stenotrophomonas maltophilia	Bacteria	[1]	WP_111190739.1	EDD
Xylella fastidiosa	Bacteria	[1]	WP_023907921.1	EDD
Shigella flexneri	Bacteria	[1]	EFY2669691.1	EDD
Pseudoxanthomonas sp.	Bacteria	[1]	WP_141453109.1	EDD
Crocinitomicaceae bacterium	Bacteria	[1]	MBT6514710.1	EDD
Flavobacteriales bacterium	Bacteria	[1]	MAC86373.1	EDD
Amycolatopsis decaplanina	Bacteria	[1]	WP_007029288.1	EDD
Amycolatopsis keratiniphila	Bacteria	[1]	WP_016332713.1	EDD
Nocardioides sp. MAH-18	Bacteria	[1]	WP_157345696.1	EDD
Rhodococcus sp. JVH1	Bacteria	[1]	WP_009475031.1	EDD
Nocardioides panaciterrulae	Bacteria	[1]	WP_218852091.1	EDD
Cryobacterium glucosi	Bacteria	[1]	WP_166791009.1	EDD
Prauserella endophytica	Bacteria	[1]	WP_137095839.1	EDD
Prauserella flavalba	Bacteria	[1]	WP_110340288.1	EDD
Longispora fulva	Bacteria	[1]	WP_203787466.1	EDD
Hoyosella altamirensis	Bacteria	[1]	WP_064440734.1	EDD
Mycobacterium tuberculosis	Bacteria	[1]	SGD88927.1	EDD
Shigella dysenteriae	Bacteria	[1]	EGE2518530.1	EDD
Hoyosella subflava	Bacteria	[1]	WP_237707834.1	EDD
Enterobacteriaceae	Bacteria	[1]	WP_001069487.1	EDD
Prauserella flavalba	Bacteria	[1]	WP_110340288.1	EDD
Tenacibaculum sp. KUL152	Bacteria	[1]	GFD89636.1	EDD
Chryseobacterium sp. ISL-80	Bacteria	[1]	MBT2704122.1	DHAD
Rhizobium leguminosarum	Bacteria	[1]	WP_129418895.1	DHAD

Escherichia coli	Bacteria	[1]	YP_026248.1	DHAD
Mycobacterium tuberculosis	Bacteria	[1]	SGC94481.1	DHAD
Anaerolineae bacterium	Bacteria	[1]	MCU0501630.1	DHAD
Nocardioides sp. Iso805N	Bacteria	[1]	WP_017934119.1	DHAD
Streptomyces cinereus	Bacteria	[1]	GGL96102.1	DHAD
Enterobacteriaceae	Bacteria	[1]	WP_001127401.1	DHAD
Shigella boydii	Bacteria	[1]	EAA4813663.1	DHAD
Chamaesiphon polymorphus	Bacteria	[1]	WP_106308272.1	DHAD
Euryarchaeota archaeon	Archaea	[3]	MBE02805.1	EDD
Candidatus Woesearchaeota archaeon	Archaea	[1]	MBM3202515.1	EDD
Euryarchaeota archaeon	Archaea	[4]	MBE01529.1	DHAD
Candidatus Nitrosopumilus salaria	Archaea	[1]	WP_008300640.1	EDD
Ferroglobus placidus	Archaea	[1]	WP_012966058.1	DHAD
Candidatus Pacearchaeota archaeon	Archaea	[1]	RLG12971.1	DHAD
Candidatus Bathyarchaeota archaeon	Archaea	[5]	HDD72113.1	DHAD
Crenarchaeota archaeon	Archaea	[6]	NPA70637.1	DHAD
Ignicoccus hospitalis	Archaea	[1]	WP_012123105.1	DHAD
Desulfurococcaceae archaeon	Archaea	[1]	MCC6057874.1	DHAD
Thaumarchaeota archaeon	Archaea	[1]	RLG07732.1	DHAD
Archaeoglobi archaeon	Archaea	[1]	MBC7109545.1	DHAD
Thermoprotei archaeon	Archaea	[1]	RLF07682.1	DHAD
Methanoregulaceae archaeon	Archaea	[1]	RPI39965.1	DHAD
Archaeoglobi archaeon	Archaea	[3]	MBC7114307.1	DHAD
Methanosarcina barkeri	Archaea	[1]	WP_048108734.1	DHAD
Archaeoglobus profundus	Archaea	[1]	WP_012940844.1	DHAD
Sulfolobus sp.	Archaea	[7]	PVU75057.1	DHAD
Sulfolobaceae	Archaea	[1]	WP_009990927.1	DHAD
Aeropyrum pernix	Archaea	[1]	WP_241759698.1	DHAD
Stygiolobus caldivivus	Archaea	[1]	WP_221290504.1	DHAD
Acidianus hospitalis	Archaea	[1]	WP_013777133.1	DHAD
Candidatus Wolframiiraptor sp.	Archaea	[1]	RLF99771.1	DHAD
Synechococcus elongatus	Cyanobacteria	[1]	WP_011243210.1	DHAD
Synechococcus moorigangaii CMS01	Cyanobacteria	[8]	MBV5257376.1	EDD
Nostoc sp. 3335mG	Cyanobacteria	[1]	PXA97457.1	EDD
Leptolyngbya valderiana BDU 20041	Cyanobacteria	[1]	OAB60313.1	EDD
Leptolyngbya sp. 15MV	Cyanobacteria	[9]	QYU66659.1	EDD
Planktothrix paucivesiculata	Cyanobacteria	[1]	WP_083620833.1	DHAD
Pseudanabaena sp. lw0831	Cyanobacteria	[1]	WP_201322407.1	DHAD
Oscillatoriales bacterium UBA8482	Cyanobacteria	[1]	HBW56505.1	DHAD
Dendronalium phyllosphericum	Cyanobacteria	[1]	WP_214433426.1	DHAD
Nostocales cyanobacterium	Cyanobacteria	[1]	TAE57107.1	DHAD
Gracilaria domingensis	Cyanobacteria	[1]	KAI0558924.1	DHAD
Phormidium sp. ETS-05	Cyanobacteria	[1]	WP_199245539.1	DHAD
Chondrus crispus	Cyanobacteria	[1]	XP_005718290.1	DHAD
Adonisia turfae	Cyanobacteria	[1]	WP_163667036.1	DHAD
Anabaena minutissima	Cyanobacteria	[1]	WP_190699939.1	DHAD
Nostoc parmelioides	Cyanobacteria	[1]	WP_190569724.1	DHAD

Trichormus azollae	Cyanobacteria	[1]	WP_013189959.1	DHAD
Nostoc sp. UHCC 0702	Cyanobacteria	[1]	WP_206268843.1	DHAD
Cylindrospermopsis raciborskii	Cyanobacteria	[1]	WP_006277049.1	DHAD
Gloeobacter violaceus	Cyanobacteria	[1]	WP_011143160.1	DHAD
Synechococcus sp. JA-2-3B'a(2-13)	Cyanobacteria	[1]	WP_011433574.1	DHAD
Pseudanabaena sp. PCC 7367	Cyanobacteria	[1]	WP_015164194.1	DHAD
Pseudanabaena sp. PCC 6802	Cyanobacteria	[1]	WP_019501081.1	DHAD
Gloeomargarita sp. SKYB120	Cyanobacteria	[1]	MCS7293203.1	DHAD
Gloeomargarita lithophora	Cyanobacteria	[10]	WP_071455593.1	DHAD
Calothrix membranacea	Cyanobacteria	[1]	WP_190617033.1	DHAD
Picochlorum sp. BPE23	Algae	[1]	KAI8114006.1	hypothetical protein
Chlamydomonas eustigma	Algae	[11]	GAX72671.1	hypothetical protein
Chlorella sorokiniana	Algae	[12]	PRW61595.1	DHAD
Polytomella parva	Algae	[1]	QKY14824.1	DHAD
Micractinium conductrix	Algae	[13]	PSC73296.1	DHAD
Coccomyxa sp. Obi	Algae	[1]	BDA41163.1	DHAD
Chlamydomonas reinhardtii	Algae	[14]	XP_001693179.1	uncharacterized protein
Volvox africanus	Algae	[15]	GIL51840.1	hypothetical protein
Ostreococcus tauri	Algae	[1]	OUS43982.1	DHAD/EDD
Micromonas pusilla CCMP1545	Algae	[1]	XP_003055297.1	predicted protein
Mougeotiopsis calospora	Algae	[16]	PRJNA849386	TSA
Mougeotia sp	Algae	[17]	PRJNA543475	TSA
Zygnema circumcarinatum	Algae	[18]	PRJNA399177	TSA
Spirogyra pratensis	Algae	[17]	PRJNA543475	TSA
Spirogyra pratensis	Algae	[19]	PRJNA242253	TSA
Spirogyra pratensis	Algae	[20]	PRJNA335946	TSA
Chara braunii	Algae	[21]	GBG73628.1	hypothetical protein
Sphagnum magellanicum	Bryophytes	[1]	KAH9533862.1	hypothetical protein
Sphagnum fallax	Bryophytes	[1]	KAH8933315.1	hypothetical protein
Physcomitrium patens	Bryophytes	[1]	XP_024389039.1	DHAD
Ceratodon purpureus	Bryophytes	[1]	KAG0623704.1	hypothetical protein
Tieghemiomyces parasiticus	Fungi	[1]	KAJ1927328.1	DHAD
Dimargaris cristalligena	Fungi	[1]	KAJ1988464.1	DHAD
Hyaloraphidium curvatum	Fungi	[1]	KAI9021181.1	DHAD
Anaeromyces robustus	Fungi	[1]	ORX87668.1	DHAD
Phlyctochytrium arcticum	Fungi	[1]	KAI9102494.1	DHAD
Thamnocephalis sphaerospora	Fungi	[1]	RKP07992.1	DHAD
Viridothelium virens	Fungi	[1]	KAF2233468.1	DHAD
Fonsecaea erecta	Fungi	[1]	XP_018695233.1	DHAD
Crucibulum laeve	Fungi	[1]	TFK40805.1	DHAD
Polychytrium aggregatum	Fungi	[1]	KAI9202125.1	DHAD
Blastomyces parvus	Fungi	[1]	PGH09273.1	DHAD
Schizosaccharomyces pombe	Fungi	[22]	NP_593729.1	Putative DHAD
Saccharomyces cerevisiae S288C	Fungi	[1]	NP_012550.1	DHAD
Elsinoe ampelina	Fungi	[1]	KAF2221669.1	DHAD
Manihot esculenta	Plant	[1]	XP_021594917.1	DHAD

Dioscorea alata	Plant	[1]	KAH7669517.1	DHAD
Nicotiana tabacum	Plant	[1]	XP_016464930.1	DHAD
Capsicum annuum	Plant	[1]	XP_016573626.2	DHAD
Populus alba	Plant	[1]	XP_034922072.1	DHAD
Jatropha curcas	Plant	[1]	XP_012092653.1	DHAD
Beta vulgaris subsp. vulgaris	Plant	[1]	XP_019104709.1	DHAD
Arachis duranensis	Plant	[1]	XP_015958659.1	DHAD
Arachis hypogaea	Plant	[1]	XP_025693829.1	DHAD
Citrus sinensis	Plant	[1]	XP_006489797.2	DHAD
Solanum lycopersicum	Plant	[1]	XP_004240214.1	DHAD
Coffea arabica	Plant	[1]	XP_027116753.1	DHAD
Glycine max	Plant	[23]	KAH1217623.1	DHAD
Catharanthus roseus	Plant	[1]	KAI5650029.1	hypothetical protein
Phaseolus vulgaris	Plant	[1]	XP_007147759.1	hypothetical protein
Salix suchowensis	Plant	[1]	KAG5227902.1	DHAD
Ziziphus jujuba var. spinosa	Plant	[1]	XP_015887506.1	DHAD
Zingiber officinale	Plant	[23]	XP_042382229.1	DHAD

DHAD, dihydroxy-acid dehydratase; EDD, 6-phosphogluconate dehydratase;
TSA, transcriptome sequence assembly

Supplementary table 2. Occurrence of EDD homologs in genomes of plastid sister groups[a]

Organism	DHAD	EDD	EDA	Reference
	Recently diverged plastid sister groups			
Gloeomargarita lithophora	WP_071455593	n.d.[b]	WP_071454108	10,24
Gloeobacter kilaueaensis	WP_023175106	n.d.	WP_023172264	24
Synechococcus elongatus	WP_208679212	n.d.	WP_208678531	24
Synechocystis sp. 6803	WP_010874288	n.d.	AGF52924	
Synechocystis sp.	WP_194072189	n.d.	WP_237269654	24
Pseudanabaena sp	WP_094530374	n.d.	PZU96335	10
Arthrospira sp.	WP_006616382	n.d.	MBS0018420	25
Lyngbya sp.	WP_265233541	n.d.	MBW4695200	25
Oscillatoria sp.	WP_272061443	n.d.	WP_017718781	25
Trichodesmium sp.	MDJ0516496	n.d.	MCL2928218	25
Prochlorococcus marinus	WP_269610147	n.d.	WP_011124409	25
Anabaena sp.	WP_066383956	n.d.	WP_190367598	25
Nodularia spumigena	WP_042202732	n.d.	WP_063872911	25
Cylindrospermopsis raciborskii	WP_071241830	n.d.	WP_009344397	25
Cyanothece sp.	NEQ98939	n.d.	WP_107670816	25
Acaryochloris marina	WP_299492222	n.d.	WP_212665323	25
Geitlerinema sp.	WP_015171406	n.d.	WP_015173050	25
Prochlorothrix hollandica	WP_026099305	n.d.	WP_017713606	25
	EDD homologs in cyanobacteria			
Nostoc sp.	WP_015141122	PXA97457	PXA92509	25
Synechococcus moorigangaii CMS01	MBV5262251	MBV5257376	MBV5257377	
Leptolyngbya valderiana BDU 20041	OAB57411	OAB60313	OAB60315	
Leptolyngbya sp.	WP_198806192	QYU66659	WP_204152276	25

[a] All sequences obtained from NCBI (https://www.ncbi.nlm.nih.gov/)

[b] n.d., not detected

Supplementary table 3. PCR primers used in this study. Primers include attB overhang for gateway cloning

Primer name	Sequence (5'→3')
GmKDPG1_attB1	GGGGACAAGTTTGTACAAAAAAGCAGGCTATGGGCACTGCAACTGCAACC
GmKDPG1_attB1_pm	GGGGACAAGTTTGTACAAAAAAGCAGGCTTCACAGTTGACAGAACCCTCTGC
GmKDPG1_attB2	GGGGACCACTTTGTACAAGAAAGCTGGGTATAAATTCTATTCACAGATTTGTTACCC
GmKDPG1stp_attB2	GGGGACCACTTTGTACAAGAAAGCTGGGTCTAATAAATTCTATTCACAGATTTGTTACCC
GmDHAD_attB1	GGGGACAAGTTTGTACAAAAAAGCAGGCTATGCAGTCCACACTCTTCAACCC
GmDHAD_attB1_pm	GGGGACAAGTTTGTACAAAAAAGCAGGCTTCGTGAAGCTGAACAAGTACAGCTCC
GmDHAD_attB2	GGGGACCACTTTGTACAAGAAAGCTGGGTCTCGTCTGTTACGCATCCAC
GmDHADstp_attB2	GGGGACCACTTTGTACAAGAAAGCTGGGTCTACTCGTCTGTTACGCATCCAC
EcKDPG_attB1	GGGGACAAGTTTGTACAAAAAAGCAGGCTTCATGAAAAACTGGAAAACA
EcKDPGstp_attB2	GGGGACCACTTTGTACAAGAAAGCTGGGTCTTACAGCTTAGCGCCTTC
EcEDD_attB1	GGGGACAAGTTTGTACAAAAAAGCAGGCTTCATGAATCCACAATTGTTA
EcEDDstp_attB2	GGGGACCACTTTGTACAAGAAAGCTGGGTCTTAAAAAGTGATACAGGT
EcDHAD-attB1	GGGGACAAGTTTGTACAAAAAAGCAGGCTTCACATGCCTAAGTACCGTTCCGCC
EcDHADstp-attB2	GGGGACCACTTTGTACAAGAAAGCTGGGTGTTAACCCCCCAGTTTCGATTTATCG

Supplementary figure 1. Amino acid alignment of representative DHAD and EDD sequences

```
DHAD_Ec    ----------------------------------------------------------------MVVIWRVLVRCGPTGM  16
DHAD_Se    -----------------------------------------------------------------MAGARALWRAT--GM  13
DHAD_Ab    -----------------------------------------------------------------MAGARGLWRAT--GM  13
DHAD_Tm    ------------------------------------------------------MRSDVIKKGLERVPHRSLLKAL--GI  24
DHAD_Syne  ---------------------------------------------------MPQYRSRTTTYGRNMAGARALWRAT--GM  27
DHAD_No    ---------------------------------------------------MPAYRSRTTTHGRNMAGARGLWRAT--GM  27
DHAD_At    MQATIFSPRATLFPCKPLLPSHNVNSRRPSIISCSAQSVTADPSPPITDTNKLNKYSSRITEPKSQGGSQAILHGV--GL  78
DHAD_Gm    MQSTLFNPTHSLIPTSPHSIRSNSGHASLSVRASIAVETP-------TETVKLNKYSSRITEPKSQGASQAVLYGV--GL  71
EDD_No     ------------------------MTVRQSIRDVTDRIAA-------RSRDSRRDYLKRIEAAREAGVYRSSLSCG--NL  47
EDD_Synm   ----------------------MPAPLHPVIEEVTARIEA-------RSRSTRAAYLSLIAEARKDGPQRKILSCG--NL  49
EDD_Wa     ------------------------MIVHPTLLAVTDRIRA-------RSQHRRDAYLKRVQAAYQSGVTRHRINCT--NI  47
EDD_Ec     --------------------------MNPQLLRVTNRIIE-------RSRETRSAYLARIEQAKTSTVHRSQLACG--NL  45
EDD_Zm     -----------------------MTDLHSTVEKVTARVIE-------RSRETRKAYLDLIQYEREKGVDRPNLSCS--NL  48
EDD_Se     --------------------------MNPNLLRVTQRIVE-------RSQQTREAYLARIEQAKTATVHRSQLACG--NL  45
EDD_Ab     -----------------------MDLPNPILAKVTERVIA-------RSQKTRSAYLQRIEHAQGKFPARGALSCA--NL  48
EDD_Agro   ------------------------MSADSRIQAITARIVE-------RSKPYRETYLERLRLQVSKGVHRSVLSCG--NL  47

DHAD_Ec    TD-------------ADFGKPIIAVVNSFTQFVPGHVHLRDLGKLVAEQIEAAGGVAKEFNTI-AVDDGIAMGHGGMLYS  82
DHAD_Se    TD-------------SDFGKPIIAVVNSFTQFVPGHVHLRDLGKLVAEQIEASGGVAKEFNTI-AVDDGIAMGHGGMLYS  79
DHAD_Ab    KD-------------EDFGKPIIAVVNSFTQFVPGHVHLKDLGQLVAAEIQAAGGVAKEFNTI-AVDDGIAMGHDGMLYS  79
DHAD_Tm    TD-------------DEMRRPFIGIVSSWNEIIPGHVHLDKVVEAVKAGVRMAGGVPFVFPTI-GICDGIAMDHRGMKFS  90
DHAD_Syne  KD-------------EDFEKPIIAVANSFTQFVPGHVHLKDLGQLVAREIERAGGVAKEFNTI-AVDDGIAMGHGGMLYS  93
DHAD_No    KD-------------SDFGKPIIAVANSFTQFVPGHVHLKDLGQLVAREIEAAGGVAKEFNTI-AVDDGIAMGHGGMLYS  93
DHAD_At    SD-------------DDLLKPQIGISSVWYEGNTCNMHLLKLSEAVKEGVENAGMVGFRFNTI-GVSDAISMGTRGMCFS 144
DHAD_Gm    SE-------------DDMAKPQVGVSSVWYEGNTCNMHLLHLSEAVRDGVAAAGMVPFRFNTV-GVSDAISMGTRGMCYS 137
EDD_No     AHGFAACTPAEKAALAGNKTLNLGIVTSYNDMLSAHQPYQFYPDIIKEAAREMGATAQVAGGVPAMCDGVTQGQPGMDLS 127
EDD_Synm   AHGFAA-SGEDKEKLKSLVWPNIAIVTAYNDMLSAHQPLGAYPDIIKAAAREAGATAQIAGGVPAMCDGVTQGQAGMELS 128
EDD_Wa     AHAYAALPVCDKGLFKAEVAPNIGIVTAFNDMLSAHQPYENYPALIKDEVRKVGATAQVAGGVPAMCDGVTQGQTGMELS 127
EDD_Ec     AHGFAACQPEDKASLKSMLRNNIAIITSYNDMLSAHQPYEHYPEIIRKALHEANAVGQVAGGVPAMCDGVTQGQDGMELS 125
EDD_Zm     AHGFAAMNG-DKPALRDFNRMNIGVVTSYNDMLSAHEPYYRYPEQMKVFAREVGATVQVAGGVPAMCDGVTQGQPGMEES 127
EDD_Se     AHGFAACQPEDKASLKSMLRNNIAIITSYNDMLSAHQPYEHYPQIIRQALHSVNAVGQVAGGVPAMCDGVTQGQDGMELS 125
EDD_Ab     AHGFAGMEDNEKLIIKVGREPNIGIVSSYNEMLSAHAPYKTFPDIIKTAARENGGVAQFAGGVPAMCDGITQGNAGMELS 128
EDD_Agro   AHGFAVCSPADKDILAGDRVPNLGIITAYNDMLSAHQPYETFPAIIRDAAKEAGGIAQVAGAVPAMCDGVTQGQPGMELS 127

DHAD_Ec    LPSRELIADSVEYMVNAHCADAMVCISNCDKITPGMLMASLRL-NIPVIFVSGGPMEAGKTKLSDQIIKLDLVDAMIQGA 161
DHAD_Se    LPSRELIADSVEYMVNAHCADAMVCISNCDKITPGMLMASLRL-NIPVIFVSGGPMEAGKTKLSDKIIKLDLVDAMIQGA 158
DHAD_Ab    LPSRDLIADSVEYMVNAHCADAMVCISNCDKITPGMLMAAMRL-NIPVVFVSGGPMEAGKVKFRGDEKAIDLVDAMVVAA 158
DHAD_Tm    LPSRELIADSIEIVASGFPFDGLVFVPNCDKITPGMMMAMGRL-NIPSVLISGGPMLAGRYN-GRDIDLITVFEAVGGYK 168
DHAD_Syne  LPSRDLIADSVEYMVNAHCADALVCISNCDKITPGMLMAALRL-NIPAVFVSGGPMEAGKVILNGEERHLDLVDAMVVAA 172
DHAD_No    LPSRELIADSVEYMVNAHCADAMVCISNCDKITPGMLMAAMRL-NIPAVFVSGGPMEAGKVVVKGKEVALDLVDAMVAAA 172
DHAD_At    LQSRDLIADSIETVMSAQWYDGNISIPGCDKNMPGTIMAMGRL-NRPGIMVYGGTIKPGHFQ-DKTYDIVSAFQSYGEFV 222
DHAD_Gm    LQSRDLIADSIETVMAAQWYDGNISIPGCDKNMPGTIIAMGRL-NRPSIMVYGGTIKPGHFE-GNTFDIVSAFQCYGEYV 215
EDD_No     LFSRDVIAMATAIALSHNMFDAAVYLGICDKIVPGLVIGALTFGHLPAVFIPAGPMPSGIPN-DEKSKVRQL------YA 200
EDD_Synm   LFSRDVIAMATAVSLSHDMFDAAICLGVCDKIVPGLLIGALRFGHLPVLFLPAGPMRSGLPN-TEKARIRQL------YA 201
EDD_Wa     LFSRDVIALSTAVALSHHVFDANLFLGVCDKIVPGLLMGALRFGHLPAVFVPAGPMTTGISN-ADKARVRQL------YA 200
EDD_Ec     LLSREVIAMSAAVGLSHNMFDGALFLGVCDKIVPGLTMAALSFGHLPAVFVPSGPMASGLPN-KEKVRIRQL------YA 198
EDD_Zm     LFSRDVIALATSVSLSHGMFEGAALLGICDKIVPGLLMGALRFGHLPTILVPSGPMTTGIPN-KEKIRIRQL------YA 200
EDD_Se     LLSREVIAMSAAVGLSHNMFDGALFLGVCDKIVPGLAMAALSFGHLPAIFVPSGPMASGLPN-KEKVRIRQL------YA 198
EDD_Ab     LFSRETIAMSTAIALSHNMFDAALCLGVCDKIVPGLLIGALQFGYLPTIFVPAGPMSSGLSN-DDKAKIRQQ------FA 201
EDD_Agro   LFSRDAIAMAAGIGLSHNMFDAAVYLGVCDKIVPGLVIAALAFGHLPAVFVPAGPMTSGLPN-DEKSRIRQL------YA 200

DHAD_Ec    DPKVSDSQSDQVERSACPTCGSCSGMFTANSMNCLTEALGLSQPGNGSLLATHADRKQLFLNAGKRIVELTKRYYEQNDE 241
DHAD_Se    DPKVSDDQSNQVERSACPTCGSCSGMFTANSMNCLTEALGLSQPGNGSLLATHADRKQLFLNAGKRIVELTKRYYEQNDE 238
DHAD_Ab    DDSYTDEEVAEFERSACPTCGSCSGMFTANSMNCLTEALGLSLPGNGSIVATHANRKKLFLKAGQLIVELAKRYYEQNDA 238
DHAD_Tm    VGKVDEETLKAIEDLACPGAGSCAGLFTANTMNSLAEALGIAPRGNGTVPAVHAKRLRMAKEAGMLVVELVKR------- 241
DHAD_Syne  DDRESDEDVATIERSACPTCGSCSGMFTANSMNCLTEALGLSLPGNGSLLATHGDRKELFLEAGRLAVKLAKQYYEQDDE 252
DHAD_No    DDSYTDEEVKTIERSACPTCGSCSGMFTANSMNCLTEALGLSLPGNGSTLATHADREKLFREAGHLIVDLAKRWYEQDDA 252
DHAD_At    SGSISDEQRKTVLHHSCPGAGACGGMYTANTMASAIEAMGMSLPYSSSIPAEDPLKLDECRLAGKYLLELLKM------- 295
DHAD_Gm    SGSINDDQRQNVIRNSCPGAGACGGMYTANTMASAIEAMGMSLPYSSSTPAEDPLKLDECRLAGKYLLELLKM------- 288
EDD_No     EGKVGRAELLEAESKSYHSAGTCTFYGTANSNQMLMEIMGLHLPGASFVNPGTPLRDALTREATKRALSLSAQ-----GN 275
EDD_Synm   EGKAGRDQLLDAESASYHSPGTCTFYGTANSNQMMMELMGLHLPGSAFINPGTPLREAMVKATARRAAKITAL-----GQ 276
EDD_Wa     EGKADREALLESEMASYHSAGTCTFYGTANSNQMLLEIMGLQLPGSSFVNPGTPLREALTRHAARLAAHMAES------K 274
EDD_Ec     EGKVDRMALLESEAASYHAPGTCTFYGTANTNQMVVEFMGMQLPGSSFVHPDSPLRDALTAAAARQVTRMTGN-----GN 273
EDD_Zm     QGKIGQKELLDMEAACYHAEGTCTFYGTANTNQMVMEVLGLHMPGSAFVTPGTPLRQALTRAAVHRVAELGWK-----GD 275
EDD_Se     EGKVDRMALLESEAASYHAPGTCTFYGTANTNQMVVEFMGMQLPGSSFVHPDAPLREALTAAAARQVTRLTGN-----GN 273
EDD_Ab     TGQVGRDALLEAESAAYHGQGTCTFYGTANSNQMLMEVMGLHLPSAAFVHPHTPLRDALTAEAAKRVLDLTAE-----RG 276
EDD_Agro   EGKVGRAELLEAESKSYHGPGTCTFYGTANSNQMLMEIMGFHMPGSSFINPGTPLRDALTREAANRALAITAQ-----GN 275

DHAD_Ec    SALP-RNIASKAXFENAMTLDIAMGGSTNTVLHLLAAAQEAEIDFTMSDIDKLSRKVPQLCKVAPSTQKYHMEDVHRAGG 320
DHAD_Se    SALP-RNIASKAAFENAMTLDIAMGGSTNTVLHLLAAAQEAEIDFTMSDIDKLSRKVPQLCKVAPSTQKYHMEDVHRAGG 317
DHAD_Ab    SILP-RSIATKAAFKNAMTLDIAMGGSTNTVLHLLAAANEAEVDFTMDDIDELSRRVPVLSKVAPAKQDVHMEDVHRAGG 317
DHAD_Tm    DVKP-RDIVTLDSFMNAVMVDLATGGSTNTVLHLKAIAESFGIDFDIKLFDELSRKIPHICNISPV-GPYHIQDLDDAGG 319
DHAD_Syne  SVLP-RSIASFKAFENAICLDIAMGGSTNTVLHLLAAAHEAGVDFTMKDIDRLSRKIPNLCKVAPSTQKYHMEDVHRAGG 331
DHAD_No    TALP-RGIATFAAFENAMSLDIAMGGSTNTVLHLLAAAQEGGVEFTMADIDRLSRRVPCLSKVAPAKSDVHMEDVHRAGG 331
DHAD_At    DLKP-RDIITPKSLRNAMVSVMALGGSTNAVLHLIAIARSVGLELTLDDFQKVSDAVPFLADLKPS-GKYVMEDIHKIGG 373
DHAD_Gm    DLKP-RDIITRKSLRNAMVIVMALGGSTNAVLHLIAIAKSVGIDLTLDDFQKVSDEVPFIADLKPS-GKYVMEDVHKIGG 366
EDD_No     DYTPIGHIIDEKAIVNGLVGLHATGGSTNHTMHLIAMAAAAGLQVTWDDMSDLSDATPLLARVYPN-GVADVNHFHAAGG 354
EDD_Synm   DYRPIGEAIDARAIINAIVGLNATGGSTNHTIHLVAIARAAGLIINWDDFEDISHVTPLLTKIYPN-GPADVNHFHAAGG 355
EDD_Wa     AALSLCDIVDERVIVNAMVGLLATGGSTNHTIHLVAIARAAGIEINWDDFSELSADVPLIARVYPN-GKADINHFHAAGG 353
EDD_Ec     EWMPIGKMIDEKVVVNGIVALLATGGSTNHTMHLVAMARAAGIQINWDDFSDLSDVVPLMARLYPN-GPADINHFQAAGG 352
EDD_Zm     DYRPLGKIIDEKSIVNAIVGLLATGGSTNHTMHIPAIARAAGVIVNWNDFHDLSEVVPLIARIYPN-GPRDINEFQNAGG 354
EDD_Se     TWMPLGKMIDEKVVVNGIVALLATGGSTNHTMHLVAMARAAGILINWDDFSDLSEVVPLMARLYPN-GPADINHFQAAGG 352
EDD_Ab     NYTPIGHVIDEKAIINGIVALLATGGSTNHTLHLIAIARAAGILIDWDDFDELSAVVPLLAKIYPN-GKADVNHFQAAGG 355
EDD_Agro   EFTPAGEMIDERSVVNGVVGLHATGGSTNHTMHLIAMARAAGIILTWQDISDLSDIVPLLARVYPN-GLADVNHFHAAGG 354
```

```
DHAD_Ec     VIGILGELDRAGLLNRDVKNVLGLTLPQTL-EQYDVML-TQDDAVKNMFRAGPAGIRTTQAFSQDCRWDTLDDDRANGCI 398
DHAD_Se     VLGILGELDRAGLLNCNVKNVLGLTLPQTL-EQYDITV-TQDEAVKKMFRAGPAGIRTTQAFSQDCRWDSLDDDRAAGCI 395
DHAD_Ab     IMAILGELDRANLLDVSVPTVHEKTLKDAL-DKWDIIR-TEDPDVYEFYRSSPGGVPTQVAFSQNRYYSTLDGDREKGVI 395
DHAD_Tm     IYAVMKRLQENGLLKEDVMTIYLRKIGDLV-REAKI-----------------------------------LNED----VI 360
DHAD_Syne   VIAILGELDRAGLLHREVPTVHSPSLGAAL-DQWDINRETATEEAKSRYLAAPGGVPTQEAFSQSKRWTALDLDRENGCI 410
DHAD_No     IMAILGELERGGLIDASQPTVHAETLGHAL-DRWDIAR-TNSDEVRNFYRAAPGGVPTQTAFSQAARWEELDTDRAGGVI 409
DHAD_At     TPAVLRYLLELGLMDGDCMTV----TGQTLAQNLENVP-SLTEGQE--------------------------------II 416
DHAD_Gm     TPAVIRYLLEQGFLDGDCMTV----TGKTLAENAELVP-PLSN----------------------------GQE----II 409
EDD_No      MGFLIRELLDEGYLHEDVKTV----WGAGL-HNYTVEA-KLIED-KLAFEPAPA--------------ESALPK----VL 409
EDD_Synm    MGFLVRELLQAGLLHGDVMTP----GADSL-ADFAREP-FLEGE-TLVWRDAPA--------------ESLDRD----VL 410
EDD_Wa      MSFLIRELLDAGLLHERVETI----LGTGL-RQWTQEP-WLDDG-QLSWRPAPA--------------TSLDAK----VL 408
EDD_Ec      VPVLVRELLKAGLLHEDVNTV----AGFGL-SRYTLEP-WLNNG-ELDWREGAE--------------KSLDSN----VI 407
EDD_Zm      MAYVIKELLSANLLNRDVTTI----AKGGI-EEYAKAP-ALNDAGELVWKPAG---------------EPGDDT----IL 409
EDD_Se      VPVLMRELLNAGLLHEDVNTV----AGFGL-KRYTLEP-WLNNG-ELDWREGAE--------------RSLDND----VI 407
EDD_Ab      VAFLIRNLLEAGLLHNDVTTV----AGKGL-QHYTKEP-KLIDG-KLTWVDGVV--------------QSLDDK----VL 410
EDD_Agro    MGFLIKQLLKQGFVHDDVRTV----FGQGL-SAYTVDA-MLDEKGAVTRQPSPE--------------QSHDPK----VL 410


DHAD_Ec     RSLEHAYSKDGGLAVLYGNFAENGCIVKTAGVDDSILKFTGPAKVYESQDDAVEAILGGKVV-AGDVVVIRYEGPK--GG 475
DHAD_Se     RSLEYAYSKDGGLAVLYGNFAENGCIVKTAGVDDSILKFTGPAKVYESQDDAVEAILGGKVV-EGDVVVIRYEGPK--GG 472
DHAD_Ab     RNAEHAFSKDGGLAVLYGNIALDGCIVKTAGVDESILKFTGSARVFESQDAAVEAILGNEIK-AGDVVVIRYEGPR--GG 472
DHAD_Tm     RPFDNPYHKEGGLGILFGNLAPEGAVAKLSGVPEKMMHHVGPAVVFEDGEEATKAILSGKIK-KGDVVVIRYEGPK--GG 437
DHAD_Syne   RDIEHAYSQDGGLAVLYGNLAEQGCIVKTAGVDENILVFSGPAVVCESQDEAVNWILNGRVK-EGDVVLIRYEGPR--GG 487
DHAD_No     RDVEHAFSKDGGLAVLFGNLAPEGCIVKTAGVDESILTFRGTARVYESQDASVAGILGNEVK-EGDVVVIRYEGPK--GG 486
DHAD_At     RPLSNPIKETGHIQILRGDLAPDGSVAKITG--KEGLYFSGPALVFEGEESMLAAISADPMSFKGTVVVIRGEGPK--GG 492
DHAD_Gm     RPVENPIKKTAHIQILYGNLAPQGSVAKITG--KEGLYFSGPALVFEGEEAMIAAISEDPSSFKGKVVVIRGEGPK--GG 485
EDD_No      TSARTPFQQTGGLKLLTGNLGR--SVIKVSAVKPEHRVVEAPARVFHGQDGLIAAFKAGELT-GDVIAVVRFSGPKAIGM 486
EDD_Synm    RPASDPIAPDGGLRVVAGTLGR--AVVKVSAVKPEHRRVSAPAVVFDTQDDLLAAFKAGRLE-RDFVAVIRFQGPKANGM 487
EDD_Wa      STVNEPFSPNGGLALLEGNLGR--GVIKLSAVAEENHVVRAPAIVFDSQQELIDAFKRGELE-RDFIAVVRFQGPRANGM 485
EDD_Ec      ASFEQPFSHHGGTKVLSGNLGR--AVMKTSAVPVENQVIEAPAVVFESQHDVMPAFEAGLLD-RDCVVVVRHQGPKANGM 484
EDD_Zm      RPVSNPFAKDGGLRLLEGNLGR--AMYKASAVDPKFWTIEAPVRVFSDQDDVQKAFKAGELN-KDVIVVVRFQGPRANGM 486
EDD_Se      ASFDKPFSPHGGTKVLSGNLGR--AVMKTSAVPVENQIIEAPAMVFESQHDVLPAFDAGLLD-RDCVVVVRHQGPKANGM 484
EDD_Ab      RSIDAPFQPDGGLRLMQGRLGR--GVIKISAVAPEHRKVKAPAIVFDSQEAVQAAFDRGELH-RDFIAVVRFQGARANGM 487
EDD_Agro    SSIETPFQSTGGLKMLTGNLGK--SVIKISAVKPERHIIEAPAIVFHDQQELQDAFKDGKLN-RDFIAVVRFQGPKANGM 487


DHAD_Ec     PGMQEMLYPTSFLKSMGLGKACALITDGRFSGGTSGLSIG-HVSPEAASGGSIGLIEDGDLIAIDIPNRGIQLQVSDAEL 554
DHAD_Se     PGMQEMLYPTSFLKSMGLGKACALITDGRFSGGTSGLSIG-HVSPEAASGGTIALIEDGDTIAIDIPNRSIQLQLNEAEI 551
DHAD_Ab     PGMQEMLYPTSYLKSKGLGKDCALVTDGRFSGGSSGLSIG-HVSPEAAEGGAIGLVEDGDTIEIDIPNRTIHLNIDDATL 551
DHAD_Tm     PGMREMLSPTSAIVGMGLAEDVALITDGRFSGGSHGAVIG-HVSPEAAEGGPIGIVKDGDLIEIDFEKRTLNLLISDEEF 516
DHAD_Syne   PGMQEMLYPTSYLKSKGLGKACALITDGRFSGGTSGLSIG-HVSPEAAEGGLIALVEQGDRIEIDIPNRRIHLAVSEEEL 566
DHAD_No     PGMQEMLYPTSYLKSKGLGKACALITDGRFSGGTSGLSIG-HVSPEAAEGGLIALVETGDPILIDIPNRVIRLDLDDAVI 565
DHAD_At     PGMPEMLTPTSAIMGAGLGKECALLTDGRFSGGSHGFVVG-HICPEAQEGGPIGLIKNGDIITIDIGKKRIDTQVSPEEM 571
DHAD_Gm     PGMPEMLTPTSAIMGAGLGKEVALLTDGRFSGGSHGFVVG-HICPEAQEGGPIGLIQNGDVINVDIKNRRIDVLVSDEEM 564
EDD_No      PELHKLTPPLGILQDRGF--KVALLTDGRMSGASGKVPAAIHMTPEAIDGGPISKIRNGDLIRLDANEGTLTFLGDEKEF 564
EDD_Synm    PELHKLTPTLGVLQDRGF--KVALVTDGRMSGASGKVLAAIHVCPEALDGGPIARVRDGDVITVCADTGVLSVDVQPDEL 565
EDD_Wa      PELHQLTPALAVLQDRGF--DVALVTDGRMSGASGKVPAAIHISPECAMGGPLARVRTGDIISLNAVHGRLDVEISEEEW 563
EDD_Ec      PELHKLMPPLGVLLDRCF--KIALVTDGRLSGASGKVPSAIHVTPEAYDGGLLAKVRDGDIIRVNGQTGELTLLVDEAEL 562
EDD_Zm      PELHKLTPALGVLQDNGY--KVALVTDGRMSGATGKVPVALHVSPEALGGGAIGKLRDGDIVRISVEEGKLEALVPADEW 564
EDD_Se      PELHKLMPPLGVLLDRRF--KIALVTDGRLSGASGKVPSAIHVTPEAYDGGLLAKVRDGDIIRVNGQTGELTLLVDEAEL 562
EDD_Ab      PELHRLTPVLGVLQDQGF--HVALVTDGRMSGASGKVPAVIHLSPEALLNGPIGKVQTGDMLIIDAEAGVLDIELDEQTW 565
EDD_Agro    PELHRLTPPLGVLQDRGF--KVALVTDGRMSGASGKVPAAIHVTPEASDCGPISLIRDGDIIRLDAISGTLEVLVSAAEL 565


DHAD_Ec     AARREAQDARGDKAWT-PK-NRERQVSFALRA-YASL---------------ATSADKGAVRDKSKLGG----------- 605
DHAD_Se     AARREAQEARGDKAWT-PK-NRQRQVSFALRA-YASL---------------ATSADKGAVRDKSKLGG----------- 602
DHAD_Ab     AHRRTIQEA---KGWH-PKEERKRKVSKALKV-YAMH---------------TTSAAKGAVRVL---------------- 595
DHAD_Tm     ERRM--------KEFT-PL-VKEVDSDY-LRR-YAFF---------------VQSASKGAIFRKP--------------- 554
DHAD_Syne   AHRRAAMEARGDQAWT-PK-DRDRPISQALQA-YAAM---------------TTSAARGGVRDLSQILGSR--------- 619
DHAD_No     AERHAKMEAKGAKAWK-PF-GRKREVSPALRA-YAAM---------------TTNAAKGAVRDVSQVERD---------- 617
DHAD_At     NDRR--------KKWTAPAYKVNRGV---LYK-YIKN---------------VQSASDGCVTDE---------------- 608
DHAD_Gm     EARR--------KKWTAPPYKANRGA---LYK-YIKN---------------VTPASSGCVTDE---------------- 601
EDD_No      FSRT--------PAIE-DLRPQQFGMGRELFAGFRNL---------------VGVADRGASVFG---------------- 604
EDD_Synm    AARA--------AAIC-DLTASHRGLGRELFAGFRQI---------------ASSAEEGALSIPGLCEPASPRPVTETVA 621
EDD_Wa      AQRE--------VATA-DLSDNQFGMGRELFSGFRGL---------------ADTAEKGCFTFAFDD------------- 606
EDD_Ec      AARE--------PHIP-DLSASRVGTGRELFSALREK---------------LSGAEQGATCITF--------------- 603
EDD_Zm      NARP-----------HAEKPAFRPGTGRELFDIFRQN---------------AAKAEDGAVAIYAGAGI----------- 607
EDD_Se      AARQ--------PHIP-DLSASRVGTGRELFGALREK---------------LSGAEQGATCITF--------------- 603
EDD_Ab      QSRP--------VAQPEHQAENEVGFGRELFGVFRAA---------------AAPAEHGASVFGALVGEEPQAHI----- 617
EDD_Agro    AKRE--------PARA-DLSGNEWGMGRELFAPSAAMPARPIRAPAFSSIDILKGEDRCGFRLSTLFCASSDCSFLPLTI 636


DHAD_Ec     ----------   -
DHAD_Se     ----------   -
DHAD_Ab     ----------   -
DHAD_Tm     ----------   -
DHAD_Syne   ----------   -
DHAD_No     ----------   -
DHAD_At     ----------   -
DHAD_Gm     ----------   -
EDD_No      ----------   -
EDD_Synm    ----------   -
EDD_Wa      ----------   -
EDD_Ec      ----------   -
EDD_Zm      ----------   -
EDD_Se      ----------   -
EDD_Ab      ----------   -
EDD_Agro    RCGNRFISHF 646
```

Supplementary figure 1. Amino acid alignment of representative DHAD and EDD sequences from prokaryotes and eukaryotes. Residues in dark and light gray represent 80% and 60% similarity cutoffs, respectively. *Ec,* Escherichia coli; *Se, Salmonella enterica; Ab; Acinetobacter baumannii; Tm; Thermotoga maritima; Syne; Synechococcus elongatus; No; Nostoc sp.; At, Arabidopsis thaliana; Gm, Glycine max; Synm, Synechococcus moorigangaii; Wa, Candidatus Woesearchaeota archaeon; Zm, Zymomonas mobilis; Agro; Agrobacterium tumefaciens*

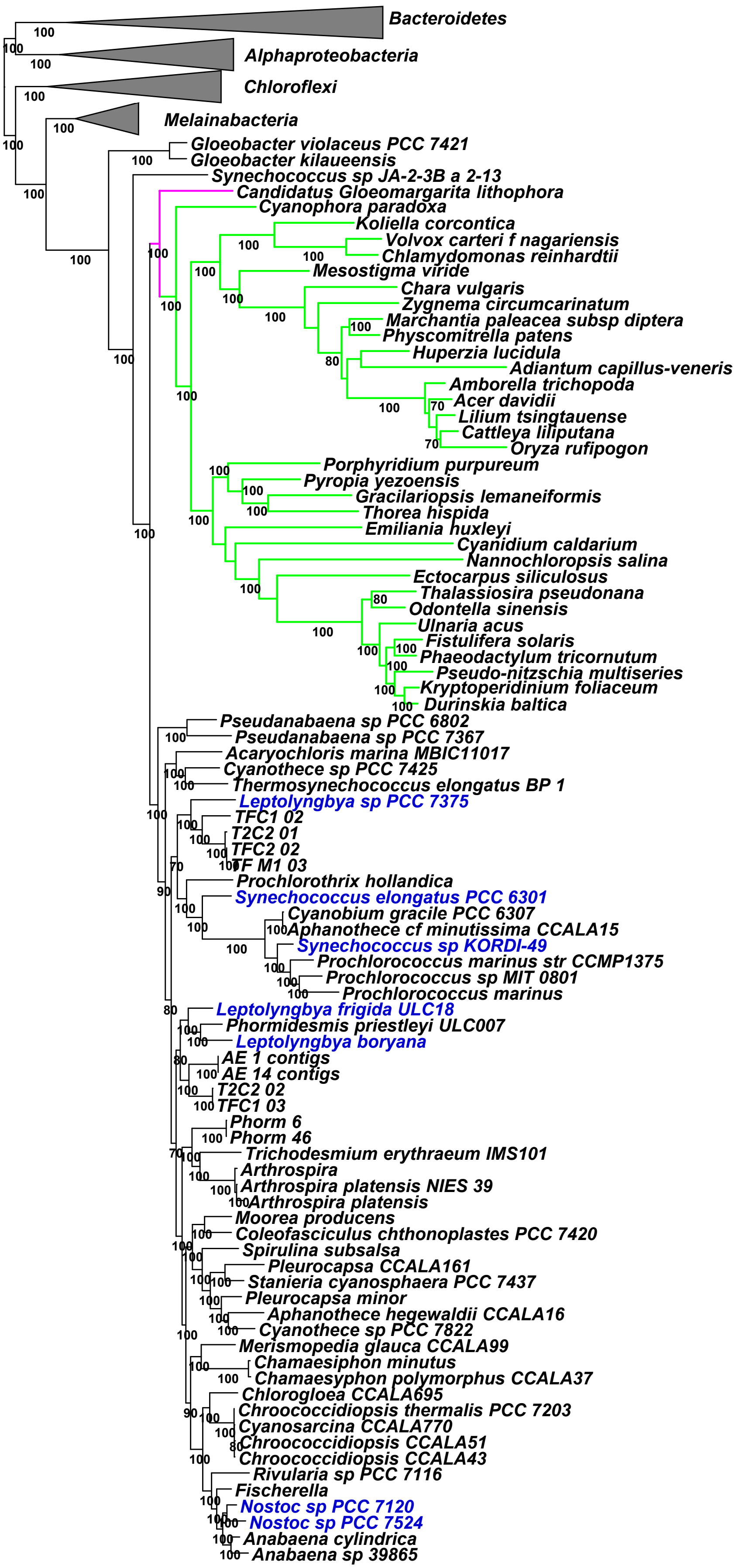

Supplementary figure 2. Species phylogeny of the Cyanobacteriota as previously reported by Moore et al.[24]. This reconstruction emphasizes species encoding both genes of the Entner-Doudoroff pathway (6-phosphogluconate dehydratase (EDD) and 2-keto-3-deoxy-6-phosphogluconate aldolase (EDA) as shown in supplemental table S2 of this study. Branch lengths in the maximum likelihood phylogenetic tree were based on concatenated ribosomal protein sequences and were downloaded from doi: 10.6084/m9.figshare.7629383. While EDA is widespread among cyanobacteria, the EDD gene is present only in a handful of species, none of which appeared in this species phylogeny. Blue text signifies their closest relatives among crown cyanobacteria. The pink branch indicates the closest sister group (*Gloeomargarita lithospora*) to the Archaeplastida (green branches).

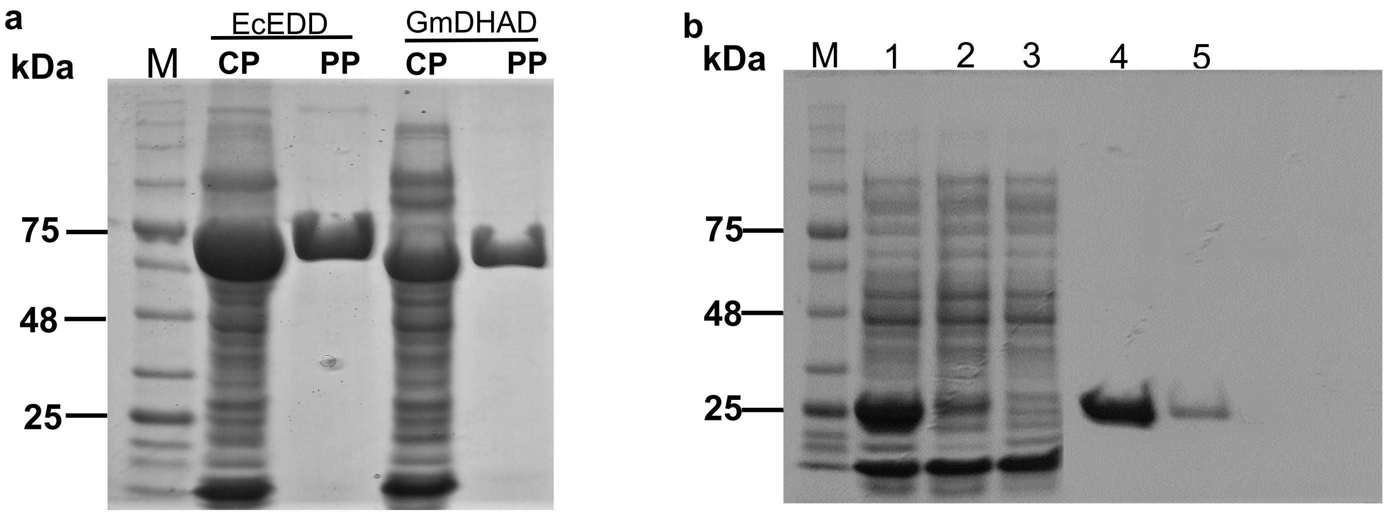

Supplementary figure 3. SDS-PAGE analysis of purified preparations of dihydroxy acid dehydratase (DHAD), 6-phosphogluconate dehydratase (EDD) and 2-keto-3-deoxy-6-phosphogluconate aldolase (EDA). Proteins from plant and bacterial sources were heterologously expressed in *Escherichia coli* BL21(DE3). **a**, Purification of *E. coli* EDD and *Glycine max* DHAD. CP, crude protein; PP, Ni-agarose purified protein. **b**. Purification of *E. coli* EDA and *G. max* EDA. 1, Crude protein extract of an IPTG-induced culture expressing EcEDA. 2, Crude protein extract of an IPTG-induced culture expressing GmEDA. 3, Uninduced control culture; 4, Ni-NTA purified EcEDA. 5, Ni-NTA purified GmEDA.

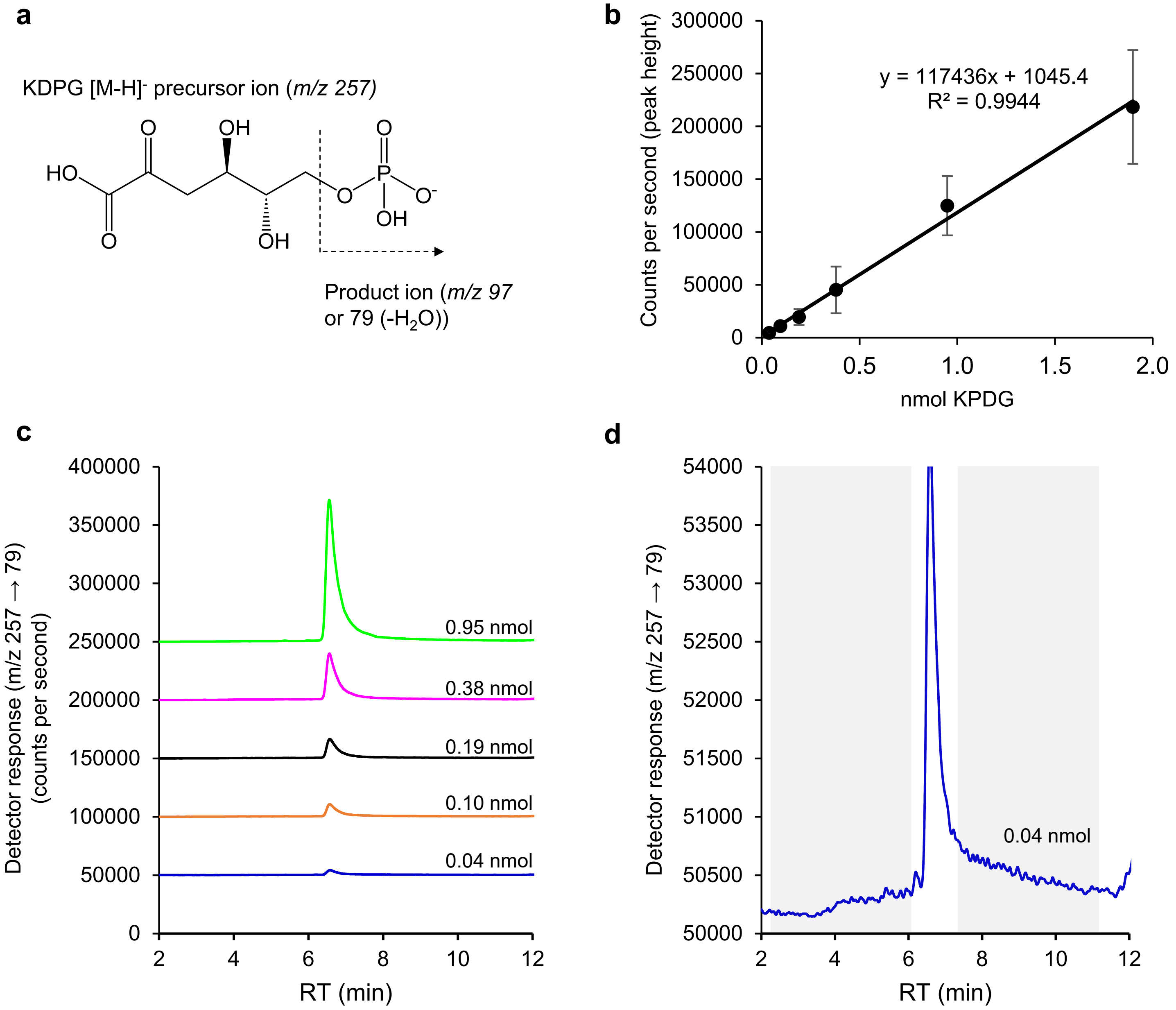

Supplementary figure 4. Analytical detection of 2-keto-3-deoxygluconate-6-phosphate (KDPG) by liquid chromatography – tandem mass spectrometry (LC-MS/MS). **a**, KDPG precursor (Q_1) and product (Q_3) ions detected by multiple reaction monitoring. **b**, Calibration curve constructed with authentic KDPG used to calculate limits of detection and quantification (LOD and LOQ) (n = 3, error bars signify standard deviation). **c**, LC-MS/MS calibration peaks of KDPG at different concentrations used to construct the curve in **b**. Each chromatogram above 0.04 nmol is vertically offset by 50,000 counts per second for improved visibility. Separation was achieved using a Waters BEH amide HILIC chromatography column (2 × 150 mm, 2.5 µm particle size). See Methods for complete analytical conditions. **d**, 100× zoom of **c** to show the region of the chromatogram used to calculate the background signal for LOD and LOQ calculations (shaded gray region). The lowest calibration level (0.04 nmol KDPG) is shown.

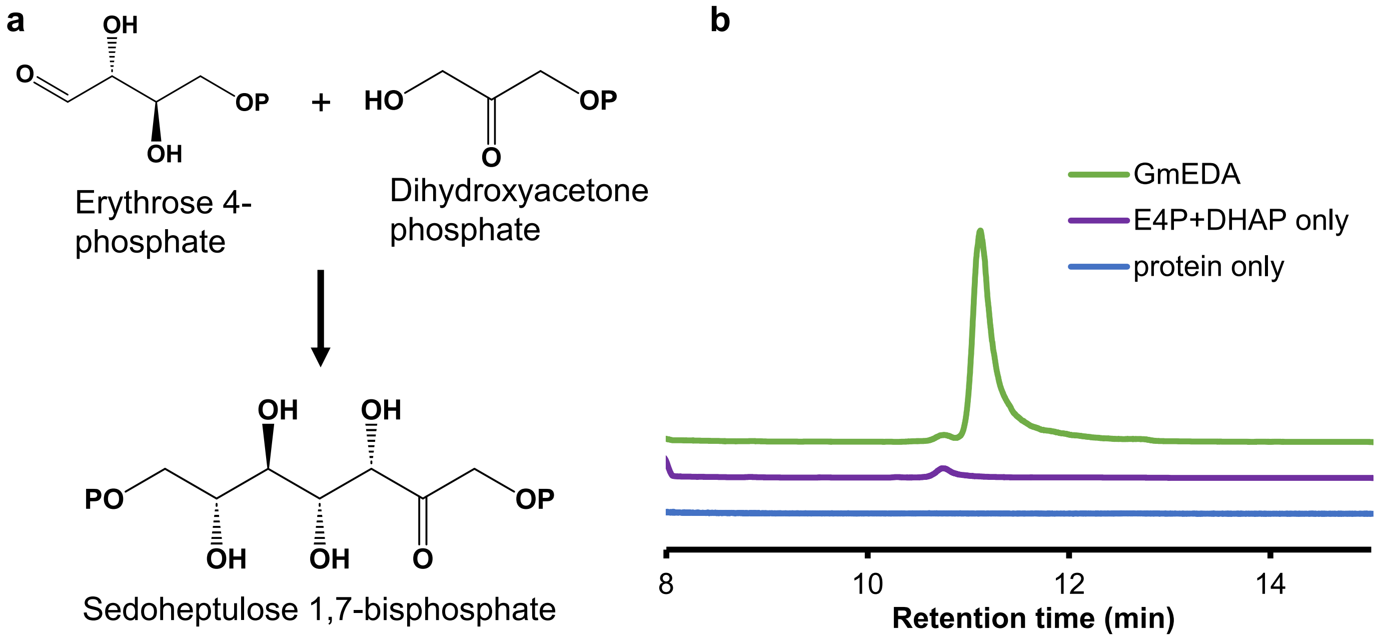

Supplementary figure 5. *In vitro* enzyme assay of purified soybean 2-keto-3-deoxygluconate-6-phosphate aldolase (GmEDA) showing aldolase activity towards substrates of the Calvin-Benson Cycle. **a**. Condensation of erythrose-D-4-phosphate (E4P) and dihydroxyacetone phosphate (DHAP) to produce D-sedoheptulose-1,7-bisphosphate (S1,7BP) as monitored by this assay (see Methods for experimental details). **b**, LC-MS/MS multiple reaction monitoring chromatogram of the GmEDA enzymatic reaction product (S1,7BP) based on its characteristic mass transition *(m/z* 369 → 79).

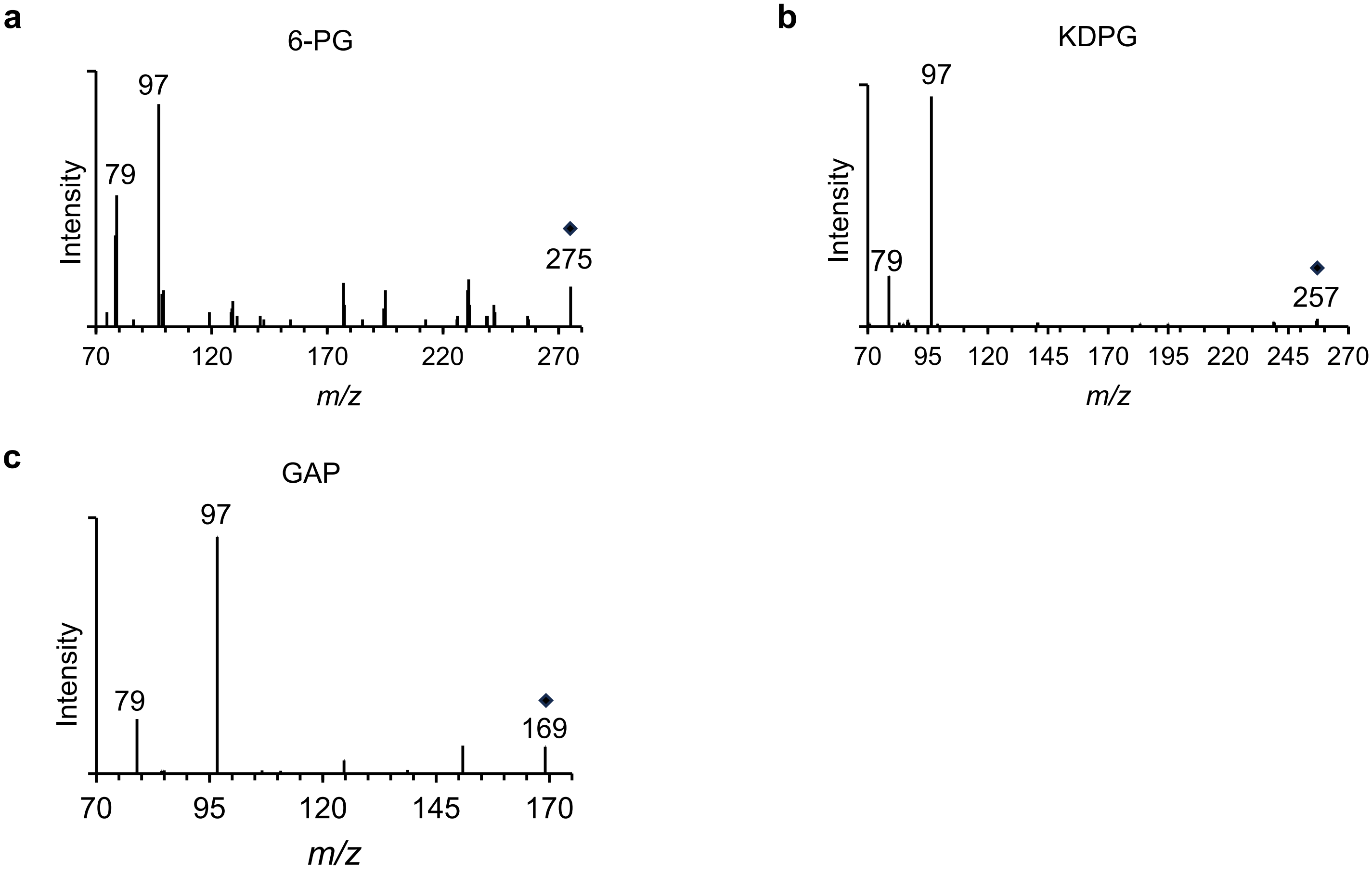

Supplementary figure 6. MS/MS product ion spectra of important metabolites used in this study. **a**, 6-phosphogluconate (6-PG); **b**, 2-keto-3-deoxy-6-phosphogluconate (KDPG); **c**, glyceraldehyde-3-phosphate (GAP). Spectra were obtained by direct infusion of authentic standards. Electrospray ionization was conducted in negative mode using nitrogen as collision gas (see Methods for additional details). The *m/z* 97 and 79 product ions represent a phosphate ion and its dehydrated phosphite form, respectively. Diamonds indicate the [M-H]- precursor ion. Each scan was repeated at least three times with identical results.

Supplementary References

1 May 2023 version of NCBI (https://www.ncbi.nlm.nih.gov/).

2 Pappas, Katherine M., et al. "Genome sequence of the ethanol-producing *Zymomonas mobilis* subsp. mobilis lectotype strain ATCC 10988." (2011): 5051-5052.

3 Liu, Yi-Fan, et al. "Anaerobic degradation of paraffins by thermophilic Actinobacteria under methanogenic conditions." Environmental Science & Technology (2020) 54.17:10610-10620.

4 Tully, Benjamin J., Elaina D. Graham, and John F. Heidelberg. "The reconstruction of 2,631 draft metagenome-assembled genomes from the global oceans." Scientific data (2018) 5:1-8.

5 Zhou, Liu, et al. "Genome-and community-level interaction insights into carbon utilization and element cycling functions of Hydrothermarchaeota in hydrothermal sediment." Msystems (2020) 5:e00795-19.

6 Hou, Jialin, et al. "Microbial succession during the transition from active to inactive stages of deep-sea hydrothermal vent sulfide chimneys." Microbiome (2020) 8:1-18.

7 Munson-McGee, Jacob H., et al. "Nanoarchaeota, their Sulfolobales host, and Nanoarchaeota virus distribution across Yellowstone National Park hot springs." Applied and Environmental Microbiology (2015) 81:7860-7868.

8 Singh, Tarkeshwar, and Punyasloke Bhadury. "Description of a new marine planktonic cyanobacterial species *Synechococcus moorigangaii* (Order Chroococcales) from Sundarbans mangrove ecosystem." Phytotaxa (2019) 393:263-277.

9 Brenes-Guillén, L., et al. "Draft Genome Sequences of Two Cyanobacteria Leptolyngbya spp. Isolated from Microbial Mats in Miravalles Thermal Spring, Costa Rica." Microbiology Resource Announcements (2021) 10.41:10-1128.

10 Ponce-Toledo, Rafael I., et al. "An early-branching freshwater cyanobacterium at the origin of plastids." Current Biology (2017) 27:386-391.

11 Hirooka, Shunsuke, et al. "Acidophilic green algal genome provides insights into adaptation to an acidic environment." Proceedings of the National Academy of Sciences (2017) 114:E8304-E8313.

12 Arriola, Matthew B., et al. "Genome sequences of Chlorella sorokiniana UTEX 1602 and Micractinium conductrix SAG 241.80: implications to maltose excretion by a green alga." The Plant Journal (2018) 93:566-586.

13 Fuentes-Ramírez, Emma O., et al. "The plastid proteome of the nonphotosynthetic chlorophycean alga *Polytomella parva*." Microbiological Research (2021) 243:126649.

14 Merchant, Sabeeha S., et al. "The Chlamydomonas genome reveals the evolution of key animal and plant functions." Science (2007) 318:245-250.

15 Yamamoto, Kayoko, et al. "Three genomes in the algal genus *Volvox* reveal the fate of a haploid sex-determining region after a transition to homothallism." Proceedings of the National Academy of Sciences (2021) 118:e2100712118.

16 Hess, Sebastian, et al. "A phylogenomically informed five-order system for the closest relatives of land plants." Current Biology (2022) 32:4473-4482.

17 De Vries, Jan, et al. "Heat stress response in the closest algal relatives of land plants reveals conserved stress signaling circuits." The Plant Journal (2020) 103:1025-1048.

18 De Vries, Jan, et al. "Embryophyte stress signaling evolved in the algal progenitors of land plants." Proceedings of the National Academy of Sciences (2018) 115:E3471-E3480.

19 Ju, Chuanli, et al. "Conservation of ethylene as a plant hormone over 450 million years of evolution." Nature plants (2015) 1:1-7.

20 Van de Poel, Bram, et al. "Transcriptome profiling of the green alga *Spirogyra pratensis* (Charophyta) suggests an ancestral role for ethylene in cell wall metabolism, photosynthesis, and abiotic stress responses." Plant Physiology (2016) 172:533-545.

21 Nishiyama, Tomoaki, et al. "The Chara genome: secondary complexity and implications for plant terrestrialization." Cell (2018) 174:448-464.

22 Wood, V., et al. "The genome sequence of *Schizosaccharomyces pombe*." Nature (2002) 415:871-880.

23 Kim, Myung-Shin, et al. "Genome assembly of the popular Korean soybean cultivar Hwangkeum." G3 (2021) 11:jkab272.

24 Moore, Magnabosco, **et al.** "An expanded ribosomal phylogeny of Cyanobacteria supports a deep placement of plastids" Frontiers in Microbiology (2019) 10:1612

25 Ochoa de Alda, Esteban, et al. "The plastid ancestor originated among one of the major cyanobacterial lineages" Nature Communications (2014) 5:4937
