# Supplementary material for: Plastid ancestors lacked a complete Entner-Doudoroff pathway, limiting plants to glycolysis and the pentose phosphate pathway
Source: Nat Commun. 2024 Feb 6;15:1102. doi: 10.1038/s41467-024-45384-y (PMC10847513; doi:10.1038/s41467-024-45384-y)
Supplement: Supplementary file 3 — Description of Additional Supplementary Files [file 41467_2024_45384_MOESM3_ESM.pdf]

Description of Additional Supplementary Files:

Supplementary Data 1: Codon usage of putative eukaryotic 6-phosphogluconate dehydratase genes
